# Malignant Pleural Effusions: Updates in Diagnosis and Management

**DOI:** 10.3390/life13010115

**Published:** 2022-12-31

**Authors:** Stephen M. Hughes, Jacob Jonas Carmichael

**Affiliations:** Department of Pulmonary, Critical Care, and Sleep Medicine, Naval Medical Center San Diego, San Diego, CA 92134, USA

**Keywords:** malignant pleural effusion, pleural disease, interventional pulmonology

## Abstract

Malignant pleural effusions remain a significant clinical problem resulting in greater than 125,000 hospitalizations per year and leading to over 5 billion dollars in healthcare utilization costs. Not only are health care expenditures related to malignant pleural effusion significant, but malignant pleural effusions also often result in significant patient discomfort and distress, largely at the end of life. Advances in management over the past several years have provided patients with greater autonomy as they are able to provide self-aid at home either alone or with family assistance. Additionally, practice changes have allowed for fewer interventions allowing patients to spend more time out of the clinic or inpatient wards.

## 1. Introduction

Malignancy is the second most common etiology for exudative effusions worldwide [1]. Malignant pleural effusions (MPEs) are most associated with solid organ etiology such as lung or breast cancer in addition to hematologic malignancies such as lymphoma. Specifically, when referring to lung cancer, nearly 50% of patients will experience an MPE at some point during their course of treatment with 15% having an effusion at the time of initial diagnosis [2]. The 2018 joint American Thoracic Society/Society of Thoracic Surgeons/Society of Thoracic Radiology (ATS/STS/STR) clinical practice guideline reports greater than 125,000 hospitalizations and over 5 billion dollars in healthcare costs related to the diagnosis and management of MPE annually [1]. While the presence of an MPE portends a poor prognosis with life expectancy typically less than 12 months from diagnosis (as short as 2–3 months in lung/GI cancers or as long as 12 months in hematologic or ovarian malignancies), recent advances in management have aided patients and their families in pursuing comfort based approaches to end of life care, providing not only relief of pleural effusion related breathlessness, but also less time in the healthcare setting [2,3]. The aim of treatment is to palliate the sensation of breathlessness associated with this pathology as management of effusions does not alter the course of the disease process. To date, these strategies have relied on the use of symptomatically driven thoracenteses, use of pleurodesis agents, and indwelling pleural catheters (IPCs). Current guidelines and updates for the use of these strategies will be discussed below [4]. 

## 2. Clinical Presentation

While anywhere from 14–41% of malignant pleural effusions are asymptomatic, MPEs most commonly present with breathlessness and can be associated with pleuritic chest pain as well. A recent meta-analysis demonstrated that simply the presence of breathlessness is an independent risk factor for mortality in patients with malignant pleural effusions [5]. Physical exam findings can range from normal to dullness on percussion, absence of fremitus, and diminished breath sounds [6]. MPEs have been found to be associated with detriments to pulmonary gas exchange, respiratory mechanics and muscle function, and hemodynamics [7]. This has been demonstrated clinically as decreased 6 minute walk test scores and reduction in mean maximum oxygen consumption per minute (VO2_MAX_) in limited prospective trials [8]. Ultimately, MPEs are the most likely eitology of massive pleural effusions (complete opacification of the hemithorax) which have rarely led to acute respiratory failure [9]. A significant portion of patients with an MPE have a previously identified malignancy, however, up to 15% of patients demonstrate pleural effusion as their presenting sign of cancer and thus MPE must be considered as an important part of the differential diagnosis in all patients presenting with a pleural effusion [10]. 

## 3. Diagnosis

### 3.1. Imaging

Because many patients who are identified to have malignant pleural effusions are asymptomatic, they are often found on routine screening or imaging completed in the evaluation of other clinical syndromes. Chest radiograph is an extremely effective means for identifying pleural effusions, able to detect as little as 200 cc of fluid in the posteroanterior (PA) or anteroposterior (AP) views and 50 cc of fluid from the lateral view. Detection is aided by the fact that only 10% of patients with MPE have less than 500 cc of fluid [11]. Alternatively, ultrasound has been identified as a more sensitive means for detecting pleural fluid in addition to further characterizing other evidence of metastatic disease by examining pleural or diaphragmatic thickening and nodularity. Ultrasound has been utilized for the detection of pleural effusion since the 1960s, however, its increased availability globally has led to a more detailed investigation into its accuracy. A 2010 systematic review by Grimberg et al. demonstrated a sensitivity of 92–96% and specificity of 88–100% for ultrasound in the detection of pleural effusion. Estimation of fluid volume has been identified to be more accurate with the use of ultrasound as well [12]. Furthermore, current 2018 ATS guidelines specifically addressed the use of ultrasound as it relates to diagnostic thoracentesis. In assessing complication rates of thoracentesis, specifically pneumothorax, the use of ultrasound guidance in localization of MPE reduced the risk from 8.9% to 1.0% (RR = 0.10, 95% CI = 0.03–0.37). As a result, a conditional recommendation was established to use ultrasound to guide pleural interventions in known or suspected MPE [1]. 

Computed tomography (CT) scans are also extremely sensitive in identifying pleural fluid. In the diagnosis of MPE, CT appears to add additional value. Improved characterization of pleural nodularity, diaphragmatic thickening, and evidence of metastatic disease in the lung, abdomen, and chest wall can further risk stratify and aid in diagnosis. Previous recommendations for CT imaging in the evaluation of unilateral pleural disease have expanded the use of CT in this diagnostic algorithm [11,13]. In 2015, Porcel et al. published their CT scan-based scoring system and validation of its accuracy. Using a 7 parameter scoring system based on the presence or absence of lung/pleural lesions, loculations, pericardial effusions, and enlarged cardiac silhouette, they were able to detect MPE with a sensitivity of 88% and specificity of 94% (Table 1) [14]. More recently, however, Reuter et al. completed a systematic review assessing the value of CT imaging for discriminating benign vs. MPE in patients with unresolved unilateral effusions. Their assessment demonstrated that while CT in evaluation of MPE remains commonly utilized, the evidence for its use as a diagnostic tool overall is weak with the available studies demonstrating significant bias in patient population and significant heterogeneity amongst the study groups [15].

Prior to 2016, the use of positron emission tomography and computed tomography (PET-CT) for diagnosis of MPE had been inconsistently utilized with systemic reviews inconclusive as to the role of this imaging modality. A recent systematic review and meta-analysis published by Fjallegaard et al. demonstrated a positive likelihood ratio of 9.9 (4.5–15.3) and negative likelihood ratio of 0.1 (0.1–0.2) of visual/qualitative image analysis of integrated PET-CT [16] Similarly to Porcel, Yang et al. developed and validated their PET-CT score for aid in diagnosis of MPE. Utilizing a scoring system based on nodular ^18^F-FDG uptake, extrapulmonary malignancy, and pleural effusion 18F-fluoro-2-deoxy-D-glucose (^18^F-FDG) uptake their scoring system was able to demonstrate a sensitivity and specificity of 83.3% (73.6–90.6%) and 92.2% (85.7–96.4%) respectively (Table 2) [17]. 

### 3.2. Diagnostic Procedures

Utilization of thoracentesis with fluid analysis according Light’s criteria (pleural fluid-to-serum protein ratio greater than 0.5, pleural fluid LDH greater than 200 IU and pleural fluid-to-serum LDH ratio greater than 0.6) [18] and subsequent pleural fluid cytological evaluation remains the gold standard initial evaluation in patients with suspected MPE. Additionally, in patients in whom serum labs are not available, pleural protein greater than 3 g per deciliter or a pleural fluid cholesterol greater than 45 mg per deciliter has also been found to be consistent with an exudative effusion [2]. Pleural adenosine deaminase (ADA) levels are routinely sent in the evaluation of exudative effusions as well. As it pertains to MPE, ADA can provide prognostic data. In a recent retrospective cohort study, pleural ADA levels less than 15 U/L and greater than 40 U/L were associated with worse survival than patients with normal levels [19]. 

The sensitivity of cytology performed on pleural effusions remains poor. A 2022 systematic review and metanalysis found an overall sensitivity of pleural fluid cytology to be 58.2% (95% CI 52.5% to 63.9%; range 20.5–86.0%) [20]. In those patients who have negative cytology following their first cytologic assessment, sending a subsequent sample is likely to increase diagnostic yield, however, this question has not specifically been evaluated in several years. A previous study demonstrated an additional 27% of patients receiving a diagnosis of MPE following a second thoracentesis, with only an additional 5% of patients detected on a 3rd assessment [21]. As a result, British Thoracic Society Guidelines recommend not repeating pleural fluid cytology following a second negative assessment. To optimize yield, as much pleural fluid should be sent for cytologic assessment with a recommendation for at least 20–40 cc. Previous studies have demonstrated volumes greater than 50 cc, however, do not further increase yield. It may be advisable if a second sample is sent to send a higher volume, however, this has not been well studied [13]. A subsequent retrospective review following the BTS guidelines demonstrated that while volumes greater than 50 cc did not increase diagnostic yield, volumes greater than 75 cc did reduce nondiagnostic and atypical results [22]. Importantly, when submitting cytology specimens, both cytologic smear and cell block assessment should be considered. Diagnostic yield appears to increase when both modalities are completed simultaneously [23].

The use of flow cytometry (FC) is infrequently used in the evaluation of nonhematologic malignant pleural effusion. Its use in characterizing lymphoproliferative disease is well established. Additional investigational methods such as immunocytochemistry and Fluorescence in situ hybridization (FISH) can further solidify the identification of hematologic malignancies [24]. As the incidence of hematologic malignancies as the primary etiology of an MPE is only 3–16% [25], the utility of flow cytometry as an initial evaluation tool has not been established while cytology remains the gold standard. A 2020 retrospective study comparing cytology and flow cytometry found similar sensitivities between the two modalities (75% and 74%, respectively) with 100% specificity in both groups. Additionally, when the two modalities were combined, sensitivity increased to 86.3%, a statistically significant increase (*p =* 0.0029). In addition to increasing yield, flow cytometry should also be considered if available locally as turnaround time improved from 5 days to 1 day when comparing FC to cytology [26]. 

In patients in whom MPE is suspected yet continue to have a negative evaluation by cytologic assessment +/− flow cytometry, current guidelines recommend tissue biopsy. Previously, closed pleural biopsies (CPB) were the main mechanism for obtaining tissue from the parietal pleura. Initial attempts at pleural biopsy utilizing the Abrams needle demonstrated a diagnostic yield of only 40% with greater than 10% risk of pneumothorax. Diagnostic yield can increase to 87% when completing CPB with CT-guidance or 76–85% with ultrasound, specifically using a cutting needle. Comparisons between CPB with CT-guidance and pleuroscopy demonstrated an increase in yield when using pleuroscopy. As a result, pleuroscopy is preferred in patients without discrete plural nodularity or mass [27]. In addition to the diagnostic yield of medical pleuroscopy demonstrating benefit over CPB with a sensitivity of 92.6%, rates of major complications are also significantly less than CPB. Only 2.3% of patients will experience a major complication to include empyema, hemorrhage, or pneumonia with death occurring in only 0.4% of patients. Additionally, following a pleuroscopy, effective pleurodesis can be completed by talc poudrage at the conclusion of the case, unlike biopsies utilizing CPB. Poudrage following pleuroscopy has demonstrated an effectiveness of 80–90% [13]. 

Recently, alternatives to the traditional forceps biopsy completed during pleuroscopy have been evaluated. A 2020 systematic review and meta-analysis was completed to assess the efficacy and safety of pleural cryobiopsy compared to forceps biopsy. This review demonstrated comparable diagnostic yields (95% for cyrobiopsy and 91% for forceps biopsy *p* = 0.019), however, mild bleeding was significantly reduced in the cryobiopsy group (67% vs. 85% *p* <0.001). Due to the requirement to obtain large portions of tissue to make an accurate diagnosis, cryobiopsy is an attractive alternative that is at least as accurate as the most commonly used current techniques [28]. 

While there has been no head-to-head comparison for pleuroscopy over video assisted thoracic surgery (VATS), there are several practical advantages of pleuroscopy. Pleuroscopy can be completed in the bronchoscopy suite by a pulmonologist and is generally less invasive than VATS which requires a thoracic surgeon and typically 2–3 entry ports. Medical pleuroscopy utilizes a single point of entry and can generally be completed comfortably with moderate sedation, not requiring anesthesia support or intubation. In addition to patient comfort, there is significant cost savings in utilizing pleuroscopy as compared to VATS. Patients with more complex pleural spaces (septations, visceral disease), on the other hand, may benefit from the increased capability of VATS performed by a thoracic surgeon [29] In performing pleuroscopy, there has been debate in the literature as to the optimal approach utilizing either rigid or a semi-rigid scope. The rigid scope offers the pulmonologist a wider variety of available tools as the diameter of the rigid scope is 9 mm as opposed to the working channel of the semi-rigid scope which is only 2.8 mm. The rigid scope offers the use of 5 mm forceps which allows for larger specimen collections. While a recent randomized controlled trial demonstrated increased diagnostic yield when utilizing the rigid scope (97.8% vs. 73.3%, *p* = 0.002), the semi rigid scope provides the advantage of familiarity with general pulmonologist and is thought to be more well tolerated [27,30]. 

### 3.3. Prognosis

The prognosis for patients found to have malignant pleural effusions is extremely poor. As noted, median survival following the diagnosis of an MPE is 3–12 months. In general, patients with a primary lung cancer have the shortest median survival while patients found to have an MPE secondary to ovarian cancer tend to have the longest survival [31]. Prior to 2014, the Eastern Cooperative Oncology Group Performance Score (ECOG) was used to aid in prognosis for patients with MPE. In 2014 Clive et al. published the results of their prognosticating scoring system (The LENT score). This was validated against ECOG alone demonstrating a statistically significant improvement in predicting survival from diagnosis of MPE. The LENT score uses LDH, ECOG, Serum neutrophil to lymphocyte ratio (NLR), and Tumor type to stratify patients into low risk, moderate risk, and high-risk categories (Table 3) [32]. In 2018, Psallidas et al. created and validated a second prognostic scoring system (PROMISE) which included hemoglobin, C-reactive protein, white blood cell count, ECOG, cancer type, pleural fluid TIMP Metallopeptidase inhibitor 1 (TIMP1), and history of previous chemotherapy or radiotherapy. Their scoring system demonstrated accurate prediction of 3 month mortality (Table 4) [33]. Most recently, a third scoring system (SELECT) was developed. This scoring system demonstrated an improved prediction model of 90-day mortality within the Asian population undergoing pleuroscopy compared to the LENT and PROMISE Score [34].

Alternatively, pH alone has been demonstrated to have predictive accuracy in prognostication. A metanalysis published in 2000 demonstrated that in patients with an MPE, a pleural pH of ≤7.28 resulted in 3-month survival of only 38.9%, whereas patients with a pleural pH > 7.28 had a 3 month survival of 61.6% [35]. More recently, a large systematic review and metanalysis was published by Peng et al. to assess prognostic biomarkers of MPE that included 82 studies and over 10,000 patients. In their study, they again demonstrated good prognostic value in the LENT score but also showed significant value in other clinical parameters (stage, distant metastasis, EFGF mutation), serum labs (Hemoglobin, albumin, CRP, VEGF), and pleural labs (pH, glucose, VEGF). Biomarkers noted to have significantly elevated hazard ratios include low pH, low glucose, high LDH, high VEGF, and high surviving [36].

## 4. Management

Following diagnosis of malignant pleural effusion, the treatment approach shifts to symptom-driven therapy. In patients who remain asymptomatic despite the presence of an MPE, current guidelines recommend no additional pleural procedures specifically relating to the MPE [1]. Repeat pleural procedures may result in fibrosis and adhesions within the pleural space resulting in difficulties encountered during procedures that may become indicated in the future. In those patients in whom their MPE appears to result in breathlessness and subsequently have improvement in symptoms with removal of fluid, several symptom-driven approaches are available.

### 4.1. Thoracentesis

The most universally available intervention for the management of MPE is simply repeat thoracentesis for symptomatic drainage. While this can effectively improve patient symptoms and is the most readily accessible intervention, there are certainly concerns with this approach. As a result of the high rate of recurrence at 1-month, current guidelines recommend against this approach if patients are expected to live beyond 1 month while others recommend using only 1 week of expected survival as metric for avoiding recurrent thoracenteses [2,31]. Despite the use of ultrasound guidance for localization of a safe procedural location, complications associated with the procedure persist. Historically, the rate of pneumothorax has been reported to be near 19%. With the wide adoption of ultrasound, that rate appears to be closer to 0–3%. Hemothorax has been associated with thoracenteses as well, however, recent literature investigating the thoracic vascular anatomy in addition to the use of ultrasound has decreased that risk to <1%. Furthermore, the often quoted guidance to hold anticoagulation or antiplatelet medications does not appear to have a significant effect on the incidence of clinically significant bleeding [37]. Finally, re-expansion pulmonary edema is an often-cited concern with large volume thoracenteses, especially in patients with known or suspected non-expandable lung (lung parenchyma that does not fully expand after the removal of pleural fluid or gas). The actual incidence of this complication is likely less than 1% as investigations into the etiology have demonstrated significantly negative intrapleural pressure is the likely etiology rather than volume of fluid removed. The use of pleural manometry (although not universally utilized and not specifically recommend with little available data) may be able to help to mitigate this complication [38]. 

### 4.2. Chemical Pleurodesis

In patients with a life expectancy greater than 1 month (1 week according to some sources), the question of more definitive intervention should be discussed. The 2018 ATS/STS/STR guidelines address the next recommended steps. In patients with MPE and expected expandable lung, the guidelines recommend either pleurodesis or indwelling pleural catheter (IPC). In those patients undergoing pleuroscopy for assistance in diagnosis, either of the procedures can be completed at the time of pleuroscopy to limit procedure time, anesthetic required, and time in the hospital or clinic. Patients receiving talc pleurodesis, however, will likely require brief hospitalization at the time of instillation [1]. This recommendation is due, in part, to the TIME2 trial published by Davies et al. comparing talc slurry pleurodesis and IPC placement. In this randomized controlled trial (RCT) they found that dyspnea improved equally in both groups at 42 days, however, there was a statistically significant improvement in patients with IPC placement at 6 months. Additionally, there was no significant difference in quality of life. While there were more adverse events in the IPC arm of the study, there was a reduction in hospitalization time within the IPC group [39]. 

Once the decision to pursue chemical pleurodesis has been made there are several options currently available. Historically talc has been the most commonly instilled agent due to its safety profile and demonstrated efficacy in multiple large studies, however, alternative agents such as bleomycin and doxycycline have been used as well [40]. Guidelines addressing mechanism for delivery allow for either chest tube placement and instillation of talc slurry or talc poudrage via thoracoscopy [4]. A 2020 randomized control trial published by Bhatnagar et al. evaluated the failure rate between the 2 delivery methods. Both arms demonstrated nearly 80% success at maintaining pleurodesis at 90 days with 1.8% (95% CI, −10.7–7.2%) less failure in the talc poudrage group, although not statistically significant [41]. 

The most feared adverse complication of talc pleurodesis remains hypoxemia and acute respiratory distress syndrome (ARDS). This appears to be secondary to the inflammatory reaction of aseptic pleuritis created during the procedure. The use of graded talc, which eliminates small particle size talc, appears to have limited the incidence of ARDS to nearly 0. Alternatively, the most common complications seen from talc pleurodesis include pain and fever in addition to pneumothorax and pneumonia. Other less commonly seen complications include re-expansion pulmonary edema, pulmonary embolism, and dysrhythmia [42].

As a result of its demonstrated efficacy talc continues to be the agent of choice. A recent systematic review and meta-analysis published by Bletsios compared talc with other chemical sclerosing agents to include doxycycline, silver nitrate, bleomycin, povidone-iodine, tetracycline, mustine, and autologous blood. Of these, talc demonstrated significantly improved pleurodesis when compared to bleomycin in the subgroup analysis. When compared to alternative chemical agents combined, talc demonstrated a significant advantage (Relative Risk: 1.26 (CI 1.13, 1.40), *p* < 0.0001) [43]. Despite the recommendation that graded talc remain the preferred sclerosing agent, availability and cost may be prohibitive in some locations. As a result, alternative agents will continue to be a necessity. Povidone iodine remains an attractive alternative. As noted, this agent has not demonstrated a statistically different efficacy when compared to talc. In 2002, Olivares-Torres et al. first demonstrated success of iodopovidone in pleurodesis of MPE with nearly 100% success in their cohort of 52 patients [44]. A 2010 RCT also demonstrated a significantly reduced hospital length of stay following pleurodesis when comparing povidone-iodine pleurodesis with thoracoscopic talc poudrage (4.5 vs. 5.7 *p* = 0.02) [45]. Amore recent systematic review and meta-analysis published by Muthu et al. demonstrated a pooled success rate of 90% with a similar success rate to other chemical agents and minimal side effects [46]. 

Per British Thoracic Society guidelines, patients receiving talc pleurodesis either via slurry or poudrage should have chest tubes removed once drainage from the tubes has decreased to less than 250 cc per day, ideally within 24–48 hours from placement of sclerosing agent [31]. In an effort to further decrease hospital length of stay, Psallidas et al. recently completed the SIMPLE RCT which utilized a scoring system based on ultrasound evidence of pleurodesis compared to standard of care based on the BTS guidelines. They were able to demonstrate a reduction in length of stay without a reduction in pleurodesis success when utilizing ultrasound as part of clinical decision making in chest tube removal [47]. Shorter hospitalization times and reduced patient discomfort with early chest tube removal may make pleurodesis an increasingly attractive option in patients that wish to avoid IPC placement. 

To assess outpatient alternatives to the typical inpatient requirements of talc pleurodesis, the IPC-PLUS trial was published by Bhatnagar et al. in 2018. In this trial, patients, with MPE and no evidence of nonexpendable lung, had IPCs placed and were subsequently randomized to talc pleurodesis vs. placebo with usual IPC drainage. 43% of patients receiving talc sustained successful pleurodesis at 35 days compared to 23% in the placebo group. Notably there was no significant difference in adverse events, hospitalizations, or catheter malfunction [3]. 

### 4.3. Indwelling Pleural Catheters

Following the ATS/STS/STR guideline statement in 2018, Iyer et al. completed a systematic review and meta-analysis specifically addressing the question of the use of IPC versus pleurodesis in the management of MPE. While there are limitations to their study, specifically the expected attrition in this population of patients, they found a statistically significant reduction in hospital length of stay and repeat pleural interventions in the IPC arm of the study (RR 0.32; 95% CI 0.18–0.55). There was, however, an increase in the risk of cellulitis (RR, 5.83; 95% CI, 1.56–21.8) but, no difference in other adverse events between the 2 study groups [48]. IPCs are specifically recommended in the situation where patients have already failed pleurodesis or are expected to or have demonstrated non-expandable lung as pleurodesis failure rates are higher in this group and these patients tend to have longer hospitalization periods [1]. 

Placement of indwelling pleural catheters comes with additional goals of care discussions with the patient and their family. In cases where ultimate removal of the indwelling catheter is sought, draining with a goal of pleurodesis, if feasible based on pulmonary anatomy, should be attempted. The 2020 American Association for Bronchology and Interventional Pulmonology (AABIP) guidelines published by Miller et al. recommend a daily draining strategy [49]. The evidence for this recommendation comes from both the ASAP and the AMPLE-2 trial. Both trials were able to demonstrate persistent autopleurodesis with daily draining as compared to a symptom driven approach. ASAP showed that the rate of autopleurodesis was significantly greater in the daily draining arm vs. a symptom driven approach with autopleurodesis seen in 47% of patients within the aggressive draining arm compared to 24% in the symptom-driven group. Additionally, they found a shorter time to diuresis between the two groups with a median time to autopleurodesis in the aggressive arm of 54 days compared to 90 days in the other group [50]. Similarly, AMPLE-2 showed a rate of autopleurodesis at 60 days of 37.2% in the daily drainage arm compared to 11.4% in the symptom-guided arm. A similar rate persisted between the 2 groups at 6 months [51]. In an attempt to improve autopleurodesis Shrager et al. recently published their data regarding the silver nitrate-coated indwelling pleural catheter as a part of the SWIFT randomized trial. The primary outcome of pleurodesis was seen in 22.1% of patients receiving a silver nitrate coated IPC compared to 32.4% of those receiving a standard IPC, not meeting predefined superiority criteria. There were no significant differences in adverse events or other quality of life metrics [52].

IPCs often remain in place for the remainder of patients’ lives and as a result are often associated with various complications. Infection, either skin and soft tissue infections or pleural space infections have been seen. Pleural infections have been seen in 0.6–12.6% of patients in addition to skin infections seen in 1.2–5.7% of patients [53]. AABIP guidelines advise in both cases to treat with antibiotics first without immediately removing the pleural catheter. In pleural space infections, fluid cultures and continuous drainage should also be attempted prior to removal of IPC. Patients with MPE are often currently receiving chemotherapy at the time that an IPC is in place. Concern has previously been raised regarding the risk of infection in this immunocompromised group and whether the placement of an IPC may subject patients to an increased risk of infection. AABIP guidelines address this concern as available data suggests no increase in IPC related infections in patients receiving chemotherapy, even in the setting of neutropenia. As a result the recommendation is leave IPCs in place unless an alternative contraindication presents [49]. 

## 5. Future Directions

Novel approaches to diagnosis and treatment continue to be evaluated. In patients with suspected but not yet clinically proven MPE, evaluation via less invasive means will continue to be a critical part of the diagnostic algorithm. Alternative biomarkers such as Nicotinamide phosphoribosyltransferase (NAMPT) is one such target of investigation. A recent study by Huang et al. demonstrated that pleural fluid NAMPT levels were significantly lower in MPE when compared to other exudative effusions [54]. Calprotectin is another such biomarker that has recently been evaluated. Botana-Rial et al. found that a pleural fluid calprotectin level <62,233.2 ng/mL had a 96% sensitivity and 60% specificity for MPE as opposed to other benign pleural effusions [55]. With respect to future treatments, multiple different agents have been recently proposed and studied, specifically agents to be injected into the pleural space. A 2022 phase 2 trial evaluating the use of intrapleural bevacizumab in patients with non-squamous, non-small cell lung cancer and MPE. In this trial, Di et al. demonstrated an objective response rate (ORR) of MPE to be 50% with the use of bevacizumab in addition to a progression-free survival of 7.0 months and significant reduction in size of malignant pleural effusion [56]. Finally, a recent systematic literature review and pooled analysis by Karampinis et al. demonstrated success in the use of intrathoracic chemotherapy. In their study they demonstrated that in patients with breast or ovarian cancer, intrathoracic chemotherapy was successful in reducing MPE in 59.1% and 87.5% of patients, respectively [57]. Multiple other chemical pleurodesis agents and indwelling catheters are currently in various stages of assessment at this time. 

## 6. Conclusions

Malignant pleural effusions significantly impact quality of life of patients with only a few months to live. Guidelines regarding the management of MPE continue to focus on restoring quality of life and patient autonomy with a goal of ensuring patients spend less time in the hospital or clinic. Indwelling pleural catheters and chemical pleurodesis continue to remain the mainstay of treatment for these patients as they have equally demonstrated quality of life benefits for patients with extremely poor prognoses.

## Figures and Tables

**Table 1:** CT Scan Scoring System for Predicting Malignant Pleural Effusions.

Parameters	OR (95% CI)	Score
**Any Pleural Lesion ≥ 1 cm**	**250 (24–2650)**	**5**
Liver metastases	30.7 (6–156)	3
**Abdominal Mass**	**15.3 (4–65)**	**3**
Lung Mass or Lung nodule/s ≥1 cm	12.2 (5–29)	2
**Absence of Pleural Loculations**	**4.3 (2–9)**	**2**
No Pericardial Effusion	23.5 (1–626)	2
**Nonenlarged Cardiac Silhouette**	**9.3 (2–48)**	**2**

CT scan score >/= 7 largely consistent with MPE. Sensitivity 88% (95% CI, 73–95%), Specificity 94% (95% CI, 83–98%), LR positive 13.8 (95% CI, 4.6–41.5), LR negative 0.13 (95% CI, 0.05–0.33). Porcel et al. [14].

**Table 2:** PET-CT Score for Diagnosing Malignant Pleural Effusion.

Parameters	OR (95% CI)	Score
**Unilateral lung nodules and/or masses with increased ^18^F-FDG uptake (SUV_max_ ≥ 2.5)**	**49.7 (10.6–233.2)**	**3**
Extrapulmonary Malignancies (primary/metastatic)	49.0 (9.8–244.3)	3
**Pleural thickening (≥3 mm) with increased ^18^F-FDG uptake (TBR > 1.8)**	**9.8 (3.0–31.0)**	**2**
Multiple nodules or masses (uni-or bilateral lungs) with increased ^18^F-FDG uptake (SUV_max_ ≥ 2.5)	3.0 (1.4–6.4)	1
**Increased Pleural Effusion ^18^F-FDG uptake (TBR > 1.1)**	**3.4 (1.2–9.6)**	**1**

A total PET-CT score of ≥ 4 points = AUC 0.949 (95% CI:0.908–0.975), Sensitivity-83.3% (73.6–90.6%), Specificity 92.2% (85.7–96.4%), PLR 10.7 (5.6–20.1), and NLR 0.2 (0.1–0.3) in diagnosing MPE. Yang et al. [17].

**Table 3:** The LENT Score Calculation.

	Variable	Score
L	LDH level in pleural fluid (IU/L)	
	<1500	0
	>1500	1
**E**	**ECOG PS**	
	**0**	**0**
	**1**	**1**
	**2**	**2**
	**3–4**	**3**
N	NLR	
	<9	0
	>9	1
**T**	**Tumor Type** **Lowest Risk Tumor Types** **#Mesothelioma** **#Hematologic Malignancy**	**0**
	**Moderate risk tumor types** **#Breast Cancer** **#Gynecologic Cancer** **#Renal Cell Carcinoma**	**1**
	**Highest Risk Tumor Type** **#Lung Cancer** **#Other Tumor types**	**2**
**Risk Categories**	**Total Score**	
Low Risk	0–1	
Moderate Risk	2–4	
High Risk	5–7	

Median (IQR) survival = Low-Risk—319 days (228–549; n = 43), Moderate Risk—130 days (47–467; n = 129) High Risk—44 days (22–77; n = 31). Clive et al. [32].

**Table 4:** The PROMISE Score Calculation.

Variable	Decision	Score
**Previous Chemotherapy**	**Positive History**	**4**
Previous Radiotherapy	Positive History	2
**Hemoglobin (g/dL)**	**≥16**	**0**
	**14 to <16**	**1**
	**12 to <14**	**2**
	**10 to <12**	**3**
	**<10**	**4**
Serum White Blood Cell Count (10^9^ cells/L)	<4	0
	4 to <6.3	2
	6.3 to <10	4
	10 to <15.8	7
	≥15.8	10
**C-reactive protein (IU/L)**	**<3**	**0**
	**3 to <10**	**3**
	**10 to <32**	**5**
	**32 to <100**	**8**
	**≥100**	**10**
ECOG Performance Status	0–1	0
	2–4	7
**Cancer Type**	**Mesothelioma**	**0**
	**All other types of cancer**	**4**
	**Lung**	**5**
TIMP1 (ng/mg protein) *	<40	0
	40 to <160	1
	≥160	2
**Total score and corresponding 3-month mortality**	**Total Score**	
<25%	0–20	
25% to <50%	21–27	
50 to <75%	28–35	
≥75%	>35	

* Optional for calculating biologic PROMISE score. Psallidas et al. [33].

## Data Availability

No new data were created or analyzed in this study. Data sharing is not applicable to this article.
